# Supplementary material for: Are carbon emissions trading and green financial instruments synergistic? -Comprehensive quantitative research based on content analysis
Source: PLoS One. 2024 Mar 7;19(3):e0298601. doi: 10.1371/journal.pone.0298601 (PMC10919601; doi:10.1371/journal.pone.0298601)
Supplement: S1 Data — (ZIP) [file pone.0298601.s001.zip › Data/地级市Z_F工作报告.zip!/╕ú╜¿/─■╡┬╩╨/└┤╫╘╣1⁄2╓┌║┼í╛┬φ┐╦╩2╛▌═°í┐_2015.docx]

各位代表：

现在，我代表宁德市人民政府向大会作政府工作报告，请予审议，并请市政协各位委员和其他列席的同志提出意见。

一、2014年工作回顾

过去的一年，在省委、省政府和市委的坚强领导下，我市各级政府深入贯彻落实党的十八大、十八届三中、四中全会和习近平总书记系列重要讲话精神，牢牢把握稳中求进工作总基调，坚持稳增长、调结构、促改革、惠民生，迎难而上、奋力攻坚，积极实施环三战略，加快推进“六新大宁德”建设，经济社会保持平稳较快发展。初步统计，全市生产总值1377.65亿元、增长10.8%；公共财政总收入140.37亿元、增长11.6%，地方公共财政收入98.92亿元、增长11.5%；全社会固定资产投资1157.99亿元、增长23.9%；出口总值36.79亿美元、增长29.6%；实际利用外资1.75亿美元、增长21%；社会消费品零售总额415.03亿元、增长12.1%；居民消费价格总水平上涨1.9%；城镇居民人均可支配收入23956元、增长9.1%；农民人均可支配收入11302元、增长11.7%。

一年来的主要工作和成效是：

（一）全力以赴稳增长，经济运行稳中有进

始终坚持发展第一要务，积极应对宏观环境变化和经济下行压力，努力保增促稳，经济发展砥砺前行。注重创新机制，加强运行调度，扎实开展“三服务”和“三千”活动，为企业排忧解难，召开民营企业创新创业大会，增强企业发展信心。注重用好用足政策，出台促进民营企业创新创业再创造等措施，实施产业转型升级行动计划和龙头促进计划，产值亿元以上企业530家，其中超百亿企业2家。注重发挥投资拉动作用，落实重大项目建设“路线图”和“五个一”等工作机制，着力挤盘子争资金，218个项目列入省科学发展跨越发展行动计划，13个大项20个子项列入中央支持福建加快发展重大项目，全年共争取转移支付资金104.34亿元，增长6.04%，有力推动固定资产投资增长。注重改善区域金融环境，加强政银企互动，设立市县应急保障资金池，着力控新化旧、增信增贷。年末金融机构本外币存贷款余额1086.27亿元、1326.16亿元，分别增长5.18%、13.18%。引进和新组建金融机构8家，在全省率先实现村镇银行县域全覆盖。

（二）坚持不懈调结构，产业基础逐步夯实

现代农业加快发展。全市农林牧渔业总产值417.67亿元，增长5.7%。粮食总产量65.62万吨，茶叶、水产、食用菌、果蔬、林竹、畜禽、中药材等传统特色产业提质增效。福安、蕉城、寿宁、周宁、福鼎被评为全国重点产茶县。各县（市、区）均创建了省级农业标准示范区、农民创业园。11个市级千亩高优农业示范园区、30个现代山地农业开发示范基地加快建设，新增各类设施农业2.6万亩,建设高标准基本农田10.88万亩。补充耕地1.49万亩，连续十五年实现耕地占补平衡。农民专业合作社总量居全省设区市首位，新增国家级农民合作社示范社13家。农村土地承包经营权确权登记颁证试点工作扎实推进。除险加固水库35座、海堤15条，治理中小河流13条。防灾减灾体系不断健全，农产品质量安全监管继续加强。扶贫开发取得实效，6个省级扶贫开发重点县争取各类帮扶资金13.22亿元，完成造福工程危房改造6000户23142人，28.4万农村居民饮水安全问题得到解决。

工业增长保持稳定。着力抓龙头、铸链条、建集群，强化工业支撑。全市规模以上工业总产值2736.78亿元、增加值649.91亿元，分别增长15.5%、15.2%。传统产业巩固提升，重点产业集群建设加快，冶金新材料、电机电器、新能源3个产业集群分别实现产值799.72亿元、571.64亿元、265.98亿元。建成1个国家中小企业公共服务示范平台和海洋、食用菌、茶叶、船舶、电机等5个产业集群服务平台。广生堂、三祥新材上市工作加快推进，白水农夫在新三板成功挂牌。创新能力有效提升，新增高新技术企业12家、省级企业技术中心5家、发明专利授权102件、驰名商标12件。

第三产业稳步发展。全年实现增加值432.28亿元、增长7.9%。商贸物流业持续发展，三都澳5万吨冷链物流、红星美凯龙等项目基本建成。旅游业加快发展，全年接待游客1601.03万人次、旅游综合收入127.49亿元，分别增长18.7%、22.5%。宁德世界地质公园顺利通过中期评估。东湖水利风景区获国家级水利风景区称号，柘荣、蕉城霍童分别被评为中国十佳生态养生旅游福地、旅游名镇。新增1个4A级景区、3个3A级景区。现代服务业加快培育，福安被评为省级电子商务示范市，古田银耳、坦洋工夫红茶在渤海商品交易所挂牌交易。

（三）强化运作促投资，项目带动成效明显

全年市级重点项目完成投资660.6亿元，投资10亿元以上的重大项目46个，完成投资321亿元。宁德核电2#机组、漳湾作业区8#、9#泊位工程、鼎信科技1780热连轧和850冷轧等85个重点项目竣工投产。京台高速宁德段、中聚天冠等一批项目加快建设。甬金、宏旺不锈钢、沈海复线福安至漳湾段、福鼎贯岭至柘荣段、屏古联络线等100个重点项目开工建设。衢宁铁路、宁德核电5#、6#机组、上白石水利枢纽工程等重大项目前期工作扎实推进。三维对接成效明显，组织赴长三角、珠三角、山东、台湾、香港等地开展招商推介，签约项目235个、总投资1395亿元。

（四）统筹兼顾抓城乡，区域发展协调推进

中心城市加快建设，累计实施城建项目88个，完成投资47.16亿元。福宁北路、和畅路等一批路网建成通车。高速公路桥下景观整治工程顺利完成，大门山公园建设加快，新增绿化面积68公顷，顺利通过国家园林城市考核命名。城区供水“一户一表”改造4800户，新增停车位390个。县域经济加快发展，福鼎、福安、柘荣、古田被评为全省县域经济发展十佳县（市），福鼎被评为福建十大醉美县城。小城镇建设扎实推进，省、市试点镇完成总体规划和一批专项规划编制，实施建设项目536个，完成投资165.51亿元。131个美丽乡村试点建设全面启动，美丽乡村一日游示范村创建工作成效初显。全面开展“两违”整治，拆除违建437.96万平方米。

（五）管控并举重治理，环境质量保持优良

坚持源头严防、过程严管、后果严惩，节能减排和重金属污染、大气污染防治持续推进，重点流域整治和农村环境综合整治不断强化。生态环境建设得到加强，造林绿化19.66万亩，治理水土流失18.81万亩，清理海上违规养殖2212亩。乱建坟墓和乱滥建寺观教堂、民间信仰活动场所专项整治工作取得阶段性成效，矿山复绿行动深入开展。县级以上集中式饮用水源水质达标率、主要流域水环境功能达标率均达100%，大气环境质量达到国家二级标准。新创建国家级生态乡镇35个、省级生态县2个、市级以上生态村50个。柘荣率先通过国家生态县技术评估。

（六）创新机制激活力，改革开放步伐加快

“申融”工作扎实推进，宁德军民融合深度发展试验区获得省里批复建设，并列入省级全面深化改革试点。行政审批制度改革进一步深化，市级审批事项精简率达53.8%。市县政府机构改革基本完成。县（市、区）行政服务中心完成标准化建设。深化工商登记制度改革，全市新增私营企业5865户，增长40.68%，市场主体突破14万户。财税体制改革深入推进，“营改增”试点范围扩大，政府购买服务试点工作启动实施，财政专项资金和政府性债务管理进一步规范。对外经贸稳步发展，外商直接投资和增资项目31项。福建电机企业欧洲服务中心成立，福安通过首个国家级出口电机质量安全示范区现场考核验收。对台交流合作持续深化，第五届电博会和陈靖姑金身巡游台湾活动成功举办。福莆宁同城化、闽东北五市一区等区域协作深入推进。

（七）集中财力办实事，人民生活持续改善

保障体系逐步健全。为民办实事项目完成38件。新增城镇就业3.24万人、城镇失业人员再就业1.08万人、农村劳动力转移就业4.19万人，城镇登记失业率控制在1.58%。高校毕业生自主创业扶持力度加大，1157人实现自主创业。城乡居民和企业职工养老保险实现转移衔接，城乡居民基础养老金最低标准、基本医疗保险政府补助标准都有新的提高，全面实施医疗救助和城乡居民大病保险。最低生活保障制度惠及13.89万人，农村低保标准提高到家庭年人均收入2100元。新建社会福利中心3个。新开工保障性住房10087套，基本建成7120套。

社会事业不断进步。教育事业优先发展，13个中小学扩容工程和17个公办幼儿园项目按序时推进，新增学位10567个。福鼎、福安顺利通过义务教育基本均衡国家评估验收。实现9所高中达标晋级。建成8个县级职教中心和16个重点专业实训基地。医疗服务水平不断提升，市医院新院区投入运营，医疗机构新增床位632张，“海云工程”新增覆盖点256个。县级公立医院综合改革全面启动，市医院、闽东医院医疗集团挂牌成立。保持适度低生育水平，人口自然增长率7.0‰。文化建设持续推进，新建65个乡镇文化信息共享服务点，新增4个国家级非遗项目、5个中国历史文化名镇名村、10个中国传统村落。国家二类城市语言文字规范化工作达标评估顺利通过。群众性精神文明创建活动持续深化。第十六届省运会成功申办。宁德籍运动员在仁川亚运会上获得一金三银。工、青、妇、残疾人工作扎实开展，统计、老区、人事编制、民族宗教、档案、老龄、地方志、气象、防震、侨务、移民等工作取得新进展。

社会保持和谐稳定。深化“平安宁德”建设，推进社会治安综合治理，加强反恐维稳工作，严厉打击各类刑事犯罪，城乡社区网格化管理不断加强，群众安全感达93.05%。深入实施“六五”普法。完善领导干部接访下访制度，积极化解信访积案，信访总量有所下降。安全生产形势保持稳定，事故发生起数和死亡人数分别下降19.4%、14.3%。福安、蕉城分别被评为全国平安渔业和平安农机示范县（市、区）。食品药品安全监管不断强化。打私、人防、双拥共建、民兵预备役和国防后备力量建设得到加强。

（八）践行宗旨转作风，自身建设切实加强

深入开展党的群众路线教育实践活动，认真落实“四下基层、四解四促”和“无会周”制度，切实解决“四风”方面存在的突出问题。认真贯彻执行中央八项规定，全市“三公”经费下降25.59%。出台《宁德市人民政府重大行政决策十条规定》，不断完善依法决策科学决策机制，建立政府与法院、工会互动联系机制，加强规范性文件合法性审查，做好行政复议和行政应诉工作。绩效管理、效能建设进一步提升。自觉接受人大及其常委会监督并报告工作，依法执行人大及其常委会决议、决定，积极配合人大常委会开展执法检查、代表视察、专项调研、工作评议和专题询问，办理市人大代表建议285件。支持政协履行政治协商、民主监督、参政议政职能，定期向政协通报情况，积极采纳委员调研成果，办理市政协提案255件。广泛听取社会各方面意见。行政监察和审计监督力度加大，廉政建设和反腐败工作扎实推进。

各位代表，过去一年，我们经受了各种挑战与考验，取得了来之不易的成绩，全市经济“稳”的基础得到巩固，“进”的态势保持向好。这是中央和省委、省政府正确领导、关心支持的结果，是在市委坚强领导下全市干部群众团结一心、奋力拼搏的结果，是市人大、市政协加强监督、鼎力支持的结果。在此，我代表市人民政府，向全市人民，向人大代表、政协委员、各民主党派、工商联、各人民团体、无党派人士、离退休老同志和社会各界人士，向中央及省属驻宁机构、驻宁部队、武警官兵、公安民警，表示衷心的感谢和崇高的敬意！

在看到成绩的同时，我们也清醒地认识到发展还面临不少困难和问题，主要有：一是产业总体实力不强，龙头骨干企业不多，企业创新能力不足，第三产业发展相对滞后；二是异地钢贸信贷风险影响不容忽视，不良贷款压降难度大，企业融资难、融资贵问题突出；三是优质教育、医疗、文化等资源总量不足，食品药品安全、社会治安、安全生产等领域还存在不少隐患；四是城乡建设和管理水平不高，“两违”现象和“脏乱差”问题比较突出；五是环境保护和节能减排压力较大，环保基础设施和环境监管能力建设有待加强；六是一些干部责任意识、担当精神不足，存在不作为、慢作为、乱作为现象，法治政府建设有待加强。我们要高度重视这些问题，采取更加有力的措施加以解决。

二、扎实做好2015年工作

今年是全面深化改革关键之年，也是“十二五”收官之年。我们要将思想和行动统一到中央对经济发展进入新常态的认识和判断上来，既要看到挑战和压力，更要看到机遇和希望，正确认识新常态、主动适应新常态、有效引领新常态，把习近平总书记关于建设新福建的要求融入到“六新大宁德”的各项事业中，贯穿于政府工作的方方面面，发挥优势、顺势而为，坚定信心、乘势而上，努力开创经济社会发展新局面。

政府工作的总体要求是：全面贯彻党的十八大和十八届三中、四中全会精神，深入学习贯彻习近平总书记系列重要讲话和来闽考察重要讲话精神，认真落实中央、全省经济工作会议要求，主动适应经济发展新常态，按照市委三届十三次、十五次全会部署，坚持稳中求进、好中求快工作总基调，以“服务发展年”、“项目落实年”、“融合创新年”为抓手，以提高经济发展质量效益为中心，全力上项目、促增长、强改革、惠民生、抓法治、保稳定，加快推进“六新大宁德”建设，为实现机制活、产业优、百姓富、生态美的新福建作出更大贡献。

今年经济社会发展的主要预期目标是：全市生产总值增长11%；公共财政总收入增长11%，地方公共财政收入增长10%；全社会固定资产投资增长25%；外贸出口增长20%，实际利用外资增长20%；社会消费品零售总额增长13%，居民消费价格总水平涨幅控制在3%以内；城镇居民人均可支配收入增长10%，农民人均可支配收入增长12%；城镇登记失业率控制在4%以内；人口自然增长率控制在9‰以内；完成单位生产总值能耗、化学需氧量、二氧化硫、氨氮、氮氧化物等年度节能减排任务。

实现上述目标，重点抓好以下八个方面工作：

（一）深化项目带动，引领科学发展跨越发展

狠抓重大项目。深入开展“项目落实年”活动，全年计划实施市级重点项目380个，总投资5096亿元，其中在建项目300个，年度计划投资527.6亿元。加快建设宁德核电3#、4#机组、中聚天冠、甬金、宏旺等在建续建项目，确保竣工90个。开工建设新能源动力电池扩能、青拓400系不锈钢、浦众不锈钢冷轧制品、鲁花食用调和油等100个项目。深化霞浦核电基地、宁德核电5#、6#机组、韩国SK系列项目前期。推行标准化管理，努力做到项目投资、进度、安全、质量同步推进，打造阳光工程、优质工程。高起点、高标准编制“十三五”规划和各类专项规划，精心谋划一批好项目大项目和重要改革举措。

深化三维对接。紧盯世界500强、全国500强、台湾前百大、行业10强，瞄准冶金新材料、电机电器、新能源等产业链核心环节和缺失项目，有针对性地开展招商引资，加快引进一批上游关键材料、核心部件和下游终端产品项目。抓住新一轮国企改革契机，加大与央属、省属企业对接力度。整合各类招商资源和平台，建立市县两级联动投资促进机制。创新电博会、投洽会市场化组织方式和招商引资方式，推行小分队招商、以商招商、网络招商等，提高招商选资实效，力争全年签约项目总投资1200亿元以上。

完善基础设施。开工建设衢宁铁路宁德段，建成合福铁路宁德段，力争铁路通车总里程突破210公里。加快沈海复线福安至漳湾段、福鼎贯岭至柘荣段、屏古联络线等高速公路建设，确保沈海复线柘荣至福安段、福寿高速、京台高速宁德段、宁连高速飞鸾至罗源段建成通车，实现县县通高速。推进一批普通国省干线公路（含镇镇有干线）建设。加快建设城澳作业区1#、2#多用途码头等5个港航项目，开工建设湾坞作业区8#、15#泊位等6个港航项目。推进上白石水利枢纽工程、官昌水库等一批重大水利项目。建成海西天然气二期管网罗源至福鼎段、霞浦闾峡风电，力争动工建设周宁抽水蓄能电站，新建扩建110千伏及以上变电站11座。

健全工作机制。深化项目前期工作，加强项目策划储备和动态管理，形成项目滚动发展态势。强化协调调度，坚持“一月一协调，一季一督查”，有效落实项目资金、用地、用林、用海等要素保障。完善政银企会商长效机制，争取扩大信贷规模，推广政府与社会资本合作的建设运营模式，充分发挥社会资本作用。优化审批服务，推行网上审批、并联审批、绿色通道办理以及重大项目全程代理，切实提高审批效率。

（二）着力增效增收，全面加强三农工作

发展特色现代农业。全面落实强农惠农政策，抓好15个万亩粮食高产创建示范片、20个500亩以上粮食产能区，加快推进省、市、区23万吨粮食储备集中区建设。重点支持茶叶、水产、食用菌、果蔬、林竹、畜禽、中药材等传统优势产业发展。大力发展设施农业、生态农业、休闲观光农业，引导农业向加工、流通环节延伸，提高农产品附加值。加快福安国家级现代农业示范区建设，提升省级农民创业园和市级千亩高优农业示范园区建设水平，继续培育市级山地农业开发示范基地，新增各类设施农业示范面积5000亩以上。推进“三品一标”认证，严格农产品质量安全监管，保障重大动植物疫情疫病防控安全。

构建新型经营体系。推进农村土地承包经营权、林权等确权登记颁证工作，鼓励承包地向专业大户、农民专业合作社流转，发展适度规模经营。做大农业龙头企业，支持龙头企业在优势产区建立农产品生产基地。引导农业企业与农户建立紧密的利益联结机制。加快培育专业种养大户、家庭农场、职业农民等新型农业经营主体，新增家庭农场50家，完成农民技能培训2万人以上。

提高扶贫攻坚实效。落实中央、省里扶持政策，推进科学扶贫精准扶贫，组织实施“六到村到户”工程，深化6个省级扶贫开发重点县、83个省市扶贫开发重点村的扶贫攻坚，改善发展条件，增强造血功能。实施造福工程危房改造5000户2万人。支持屏南县开展全国农村改革试验区扶贫开发综合改革试验工作。推动老区基点村、少数民族聚居区、海岛、库区等地区加快发展。

（三）致力创新转型，打造升级版产业

加快壮大产业集群。以龙头带动产业提升，加大对龙头企业的政策扶持和要素保障力度，鼓励支持龙头企业兼并重组，延伸上下游产业链，加快打造冶金新材料、电机电器、新能源3个千亿产业集群，力争规模以上工业总产值突破3100亿元。以园区吸引产业集聚，完善园区基础设施，提升综合承载能力，重点推进海西宁德工业区、漳湾和湾坞不锈钢产业片区、闽浙边贸工业园和“一县一园区”规划建设。以服务助力企业发展，加强工业运行调度，深入实施“一业一策”、“一企一策”，帮助企业解决生产经营中的困难和问题。推动企业上市，支持一批企业进入海峡股权交易中心、新三板挂牌交易，鼓励企业发行公司债券、短期融资券、中小企业集合票据，提高直接融资水平。

大力培育现代服务业。加快建设大润发商场和闽东现代物流园等一批商贸（物流）项目，尽快实现红星美凯龙、盛辉物流开业运营。推进普惠金融体系建设，支持在宁金融机构扩大规模、拓展业务、创新产品，规范发展保险和证券业务。着力控新化旧、增信增贷，落实金融支持实体经济政策，积极防范和化解金融风险。大力发展电子商务，引进和培育一批电商企业，打造食用菌、水产、按摩器、茶叶等网上专业市场和行业垂直电商平台。加快发展旅游产业，打造精品景点和线路，积极培育滨海旅游、乡村旅游、文化旅游、生态旅游等多种业态。办好宁德世界地质公园文化旅游节。落实稳定住房消费政策，促进房地产市场健康平稳发展。鼓励发展健康、养老、家政、社区等服务。

着力发展海洋经济。积极发展临港工业、港口物流、海洋工程装备和海洋生物医药产业，加快宁德国家农业科技园区核心区、大黄鱼产业园区建设。逐步推广高位池养殖和工厂化循环水养殖。着力培育水产品专业批发交易市场和冷链物流企业。大力发展水产品精深加工，打造知名品牌。加快沙埕、三沙中心渔港等一批渔港建设，带动渔区、渔村发展。推动已获批建设的14艘远洋渔船尽快投产。

切实强化创新驱动。积极促进互联网与工业融合发展，实施一批信息化工业化融合示范项目。落实固定资产加速折旧、研发费用加计扣除等鼓励创新政策，支持企业承担重大科技专项等创新项目。推动高校与科研院所来宁德设立研发机构，加快5个科技企业孵化器建设。抓好高性能伺服电机科研攻关，配套推进“数控一代”机械产品创新应用示范工程。推进100个投资3000万元以上重点技改项目，力争技改投资增长10%以上。认真落实省“海纳百川”高端人才聚集计划，加大高层次人才引进和培养力度。

（四）坚持城乡统筹，扎实推进新型城镇化

做强中心城市。加强规划引导，探索“多规合一”，实施“东扩面海，北展南移”战略，推动金涵、金塔、东兰组团集中发展，加快推进滨海大道及其内侧围填海项目建设。有序拓展城区路网，动工建设金漳路、天德路、金马北路，建成闽东中路。进一步完善市政配套设施，新建改建污水管网98公里、供水管网102公里、燃气管网17公里，改造“一户一表”5000户。动工建设东区污水处理厂、漳湾污水处理厂、市餐厨垃圾处置中心和污泥处置中心。实施一批园林绿化项目，改造中心城区6大公园和4条绿道，切实提升中心城市景观。

做大县域经济。立足资源禀赋、产业基础，凸显县域特色，探索差异化发展路径。加快实施“大城关”战略，推进新区组团式发展、老城区有序改造。坚持以城促产、强产兴城，结合“一县一园区”建设做大“一县一业一品”，打造县域特色经济。完善沿海与山区产业联动发展机制，加强山海协作，落实对口帮扶政策，促进省级扶贫开发重点县共建园区加快建设。支持福安、屏南开展省级新型城镇化试点工作。

做精特色城镇。提升城镇发展规划，优化镇乡空间布局，支持有条件的中心镇与周边乡镇连片融合发展。加快省市试点镇发展，结合各地特色优势，增强产业实力，完善市政配套，建设一批城镇道路、园林绿化和污水垃圾处理项目，提高城镇综合承载能力和管理服务水平。推进赛岐镇、太姥山镇小城市试点，落实简政放权、强镇扩权，探索镇级市改革发展的新体制新机制。

做美城乡环境。深入实施宜居环境建设行动计划，延伸拓展“点线面”攻坚，全面推进农村生活垃圾治理，提升城乡“三边三节点”整治和美丽乡村建设水平。强化城市管理综合执法，大力整治“脏乱差”、交通秩序混乱、违章占道经营等行为。继续深化“两违”专项治理，巩固提升“两乱”整治成果。加强社区建设，提升物业管理水平。继续实施公交优先、村村通客车、农村公路养护、海岛交通便民工程，改善城乡居民出行条件。

（五）突出先行先试，进一步深化改革开放

深化重点领域改革。组织实施“融合创新年”，完成军民融合试验区、三都澳军民融合现代化深水大港等规划编制，启动宁德（福安）军民融合产业园建设。深化行政审批制度改革，继续下放、委托一批审批事项，确保实现年末除法律法规明令不得下放、上级委托不同意下放事项外，市级基本不再直接履行行政审批职能的目标。深化医药卫生体制改革，探索建立分级诊疗制度，推进公立医院综合改革全覆盖，推动市医院、闽东医院医疗集团稳步发展。深化财税体制改革，实行全口径预算编制，推进预算公开，完善财政性资金管理，加强政府债务规模控制。强化政府投资项目和资金的管理，结合行政职能转变，逐步扩大政府购买服务范围。落实“营改增”等结构性减税政策，不断优化财源结构。完善国有资产管理体制，提高市属国有企业竞争实力和筹融资能力。稳步推进事业单位分类改革、机关事业单位工作人员养老保险制度改革。稳步实施户籍制度改革，有序放开县城及建制镇落户限制。

积极扩大对外开放。深化口岸通关便利化工作，落实“三个一”通关模式，促进三都澳港区扩大开放、漳湾作业区8#、9#码头临时开放和三沙、沙埕港区开放，加快白马港区口岸联检中心建设。支持古田申报侨商投资实验区。推进重点出口行业转型升级，培育外贸综合服务企业，支持农水产品检测中心、跨境电子商务等外贸公共服务平台建设，继续鼓励和引导企业设立境外营销服务网点及研发机构。推进出口大黄鱼、茶叶、食用菌3个质量安全示范区建设。主动融入“21世纪海上丝绸之路”发展。

加强宁台交流合作。深化宁台产业合作，推动三沙、沙埕开展对台贸易。办好第七届海峡论坛?陈靖姑文化节和海峡两岸各民族欢度“三月三”节暨福建省第四届“三月三”畲族文化节，推动临水宫争创“海峡两岸交流基地”，加强圆瑛、甘国宝、马仙、畲族文化对台交流。推动宁德师院、宁德职业技术学院与台湾高校开展学生互换、教师互访、学术互通活动。

（六）推进绿色发展，加快生态文明先行示范区建设

加大生态创建力度。深入开展省级生态市和国家环保模范城市创建活动，力争省级生态县、国家级生态乡镇创建率均达80%以上，省级生态市和中心城市、福安、福鼎创模指标全面达标。划定生态功能红线，制定重点流域生态补偿办法，加大限制开发区、重点生态功能区、流域上游地区的保护和补偿力度。整治城区内河（湖），加强滨海、河流湿地保护，抓好矿山生态恢复。继续推进“四绿工程”，加强生态景观建设，实施造林绿化14.4万亩、森林抚育19万亩，完成水土流失治理15万亩，务必保护好我们的青山绿水。

强化环境综合整治。认真落实“河长制”，实行县域交界断面水质预警考核，推进城乡污水处理厂和工业园区污水集中治理设施建设，深化石板材、拆修造船、畜禽养殖等重点行业污染治理，提高重点流域和水源保护区综合整治实效。建立中心城市空气环境质量预测预报和县（市、区）自动监测系统，开展燃煤锅炉、扬尘粉尘、机动车尾气等专项整治，实施福鼎、霞浦合成革集中区热电联产集中供热工程。完成危废处置中心建设并投入运行，强化重金属、核与辐射环境监管。深入实施环境监管能力建设三年行动方案，严厉打击环境违法行为。

节约集约利用资源。落实耕地保护政府目标责任制，严守耕地保护红线，从严控制占用耕地，推进土地整治，实现耕地占补平衡。鼓励开发利用地下空间，合理开发低丘缓坡地。提高海域、岸线等资源节约集约利用水平，组织清收长期闲置的海域使用权。科学开发利用水资源，大力推广节水技术和产品。实施20个重点节能项目，强化工业、建筑、交通运输、公共机构节能措施，确保实现年度节能目标。

（七）办好惠民实事，促进发展成果更多惠及人民

提升社会保障。加强基层就业和社会保障服务设施建设，促进高校毕业生就业创业，多渠道实现就业困难人员、被征地农民和失业人员就业再就业。农村居民最低生活保障标准提高到2300元。城乡居民基本医疗保险政府补助标准提高到380元。推动新型农村合作医疗、城镇职工和居民基本医疗保险等经办机构整合，促进资源优化配置。加快金涵小区保障房建设，推进廉租房、公租房并轨运营，加强棚户区（危旧房）改造，保障性安居工程新开工5769套、基本建成3195套。推进市县两级社会福利中心建设和运营，加大非营利性民办养老机构扶持力度。做好关心下一代工作，重视发展慈善、老龄、红十字和残疾人事业。

优先发展教育。落实第二轮学前教育三年行动计划，新建16所公办幼儿园，新增学位4500个。推进义务教育均衡发展，实施“大手牵小手”帮扶薄弱学校建设五年行动计划和城区中小学扩容增量工程，新增学位3500个。推动古田、柘荣、寿宁、霞浦通过义务教育基本均衡省级评估。整合优化普通高中，提高办学质量。创新现代职业教育人才培养模式，力争中职学校毕业生“双证书”率达95%以上。推进宁德师院三期建设，做好教育部本科教学工作合格评估迎检工作。办好继续教育、特殊教育、民族教育和老年教育。全面实施“名师学科带头人和骨干教师培养工程”，加强师德师风建设。

加强卫计服务。抓好市级重点专科和县级医疗机构建设，强化基层卫技人员教育培训，推行乡村（社区）医生签约服务。持续推进“海云工程”。实施乡村医生养老保险制度，逐步建立乡村医生医疗责任险。加强疾病预防控制、妇幼健康、卫生监督、卫生应急等工作。基本公共卫生服务经费人均标准提高到40元。强化出生人口性别比专项整治，创造良好人口环境。

推进文化建设。加强志鉴地情资料编研和历史文化名镇名村、历史建筑、传统村落保护性开发利用，加快创建省级畲族文化生态保护示范点。推动文化与科技、旅游、金融深度融合，加快文化创意产业园建设，壮大一批文化龙头企业。繁荣发展文艺创作。常态化开展文化惠民活动。积极筹办、备战十六届省运会和十届省老健会，推进体育场馆和综合配套设施建设。办好首届全国青运会击剑比赛。大力开展全民健身运动。

（八）创新社会治理，保障人民安居乐业

加强社会服务管理。积极培育和践行社会主义核心价值观，加强公民思想道德建设，健全社会信用体系，深化文明城市、文明村镇和文明单位创建。按时完成新一届村（居）换届选举工作，加强城乡社区和重点乡村网格化建设。加快“数字宁德”和智慧城市建设，完善电子政务公共服务平台。引导各类社会组织健康发展，支持工会、共青团、妇联等人民团体发挥桥梁纽带作用。

有效化解社会矛盾。深化“平安宁德”建设，加快创新立体化社会治安防控体系，严厉打击各种刑事犯罪。完善社会稳定风险评估和突发事件应急处置机制，重视新媒体运用和管理，提高网络舆情研判导控处置能力。进一步畅通信访渠道，深化领导干部接访下访活动。深入开展法治宣传教育，推进公共法律服务体系建设，完善司法救助和法律援助制度。全面落实党的民族政策和宗教工作方针，促进民族团结、宗教和顺。

切实维护公共安全。严格落实安全生产“党政同责、一岗双责”要求，强化企业主体、部门监管和属地管理责任。全面实施安全生产标准化建设三年提升工程。加强危险化学品、消防、渔业渔船、道路交通安全等重点领域专项整治，坚决防范和遏制重特大事故发生。强化食品药品安全监管，持续治理“餐桌污染”，建设“食品放心工程”。推进气象现代化建设，健全防灾减灾救灾体制，加快水资源管理系统和防汛指挥中心建设。落实县（市、区）山洪灾害防治项目，继续实施赛江流域、闽江古田溪（古田段）防洪工程。抓好30座病险水库、15条海堤除险加固，解决5.18万农村居民饮水安全问题。加强国防动员、民兵预备役、双拥共建和海防、人防、打私等工作。

三、加强政府自身建设

各位代表！新的形势、新的任务对政府自身建设提出了新的更高要求，我们要坚持全心全意为人民服务，牢固树立法治意识，执政为民，清正廉洁，努力建设人民满意的政府。

坚持依法行政，着力建设法治政府。依法全面履行政府职能，推行政府权力清单制度，推进政府事权规范化。健全依法决策机制，实施重大行政决策十条规定，逐步推行政府法律顾问制度。加强市县政府法制机构和队伍建设。深化行政执法体制改革，健全行政执法和刑事司法衔接机制，推动县（市、区）开展相对集中行政处罚权工作。落实行政执法责任制，做到严格规范公正文明执法。强化行政权力制约和监督，依法接受人大及其常委会的法律监督和工作监督，主动接受政协民主监督，认真听取各民主党派、工商联、人民团体和无党派人士意见，提高人大代表建议、意见和政协提案办理质量。全面推进政务公开，实现决策、执行、结果公开透明，增强政府公信力。

坚持改进作风，着力建设服务型政府。巩固扩大党的群众路线教育实践活动成果，坚决反对“四风”，形成改进作风常态化机制。深入开展“服务发展年”活动，提高政策研究能力、创新运作能力、攻坚协调能力和行政执行能力。坚持“四下基层”和“一线工作法”，落实“马上就办”工作机制，深化“三千”活动，切实帮助基层、帮助企业、帮助群众协调解决实际问题。发扬钉钉子精神，坚持说了算、定了干，对确定的目标、制定的政策、部署的任务，一抓到底，务求实效。加强绩效管理，严格效能问责，严明纪律，确保政令畅通。

坚持清正做事，着力建设廉洁政府。严格落实党风廉政建设责任制，坚持用制度管权、管事、管人，把权力关进制度的“笼子”，从源头上预防和治理腐败。强化廉洁自律，守住做人、处事、用权、交友的底线，老老实实干事、清清白白做人。广大公务人员要切实做到心中有党、心中有民、心中有责、心中有戒。认真落实中央八项规定，严格执行财经纪律，严控“三公”经费。加强行政监察和审计监督，坚决纠正不作为、乱作为，坚决克服懒政、怠政，坚决惩处失职、渎职，加大惩治腐败力度，努力形成干部清正、政府清廉、政治清明的良好风尚。

各位代表！新机遇孕育新希望，新常态要有新作为。让我们紧密团结在以习近平同志为总书记的党中央周围，在省委、省政府和市委的坚强领导下，紧紧依靠全市人民，抢抓机遇，开拓进取，凝心聚力，真抓实干，为加快建设“六新大宁德”而努力奋斗！
